# Hepatotoxic Components Effect of *Chebulae Fructus* and Associated Molecular Mechanism by Integrated Transcriptome and Molecular Docking

**DOI:** 10.3390/molecules28083427

**Published:** 2023-04-13

**Authors:** Liwen Ai, Fan Yang, Wanjun Hu, Liyang Guo, Weixue Liu, Xuexue Xue, Lulu Li, Zunlai Sheng

**Affiliations:** 1 College of Veterinary Medicine, Northeast Agricultural University, Harbin 150030, China; 2 Heilongjiang Key Laboratory for Animal Disease Control and Pharmaceutical Development, Harbin 150030, China

**Keywords:** *Chebulae Fructus*, differentially expressed genes, hepatotoxicity, lipid metabolism, integrated transcriptome

## Abstract

*Chebulae Fructus* (CF) is a natural medicinal plant widely used for its various pharmacological properties. Natural products used to cure several diseases have been considered safe thanks to their little or no side effects. However, in recent years, a hepatotoxic effect has been found due to the abuse of herbal medicine. CF has been reported to have hepatotoxicity, but the mechanism is unclear. In this experiment, the toxic aspect and mechanism of CF action were evaluated by transcriptome analysis. Components of toxic CF fractions were identified by LC-MS, and hepatotoxic toxic components in toxic CF fractions were predicted by molecular docking. The results showed that the ethyl acetate part of CF was the main toxic fraction, and transcriptome analysis found that the toxic mechanism was highly related to lipid metabolism-related pathways, and CFEA could inhibit the PPAR signaling pathway. Molecular docking results showed that 3′-*O*-methyl-4-*O*-(n″-*O*-galloyl-β-d-xylopyranosyl) ellagic acid (*n* = 2, 3 or 4) and 4-*O*-(3″,4″-*O*-digalloyl-α-l-rhamnosyl) ellagic acid have better docking energies with PPARα protein and FABP protein than other components. In summary, 3′-*O*-methyl-4-*O*-(n″-*O*-galloyl-β-d-xylopyranosyl) ellagic acid (*n* = 2, 3 or 4) and 4-*O*-(3″,4″-*O*-digalloyl-α-l-rhamnosyl) ellagic acid were the main toxic components, which may play a toxic role by inhibiting the PPAR signaling pathway and affect lipid metabolism.

## 1. Introduction

Natural products have long been considered good alternatives to Western medicines because of their low or no side effects and broad-spectrum activity and are widely used to treat a variety of diseases, such as cancer, heart disease and liver disease [1,2,3]. However, in recent years, evidence of the toxic effects of traditional herbal medicine has started to accumulate; thus, scientists felt the responsibility to investigate them, especially the effects on the liver, since the damage caused by the abuse of herbal medicine has become a growing concern [4].

*Chebulae Fructus* (CF) is the dried ripe fruit of *Terminalia chebula Retz.* (Combretaceae) was included in the Chinese Pharmacopoeia in 2015 and is widely used to relieve persistent diarrhea and dysentery, prevent blood in the stool and treat rectal prolapse, etc. It is rich in several different vitamins, minerals and proteins, which are actually representing the main content in the majority of herbal preparations [5]. CF exerts a protective effect against acute liver injury [6,7,8] due to its various antioxidant polyphenols. On the other hand, hydrolyzable tannins contained in CF can cause liver injury [9]. Thus, the hepatotoxicity of CF limits its use in clinical practice. However, so far, only a few studies have focused their attention on the hepatotoxic effects of CF. Therefore, considering the wide application of traditional herbal medicine in clinical practice, it is essential to carry out basic toxicological research to provide scientific data for its clinical application.

Based on the above studies, in preliminary experiments, we found that CF has a liver-damaging effect. To our knowledge, there are no reports on the mechanism of action of CF hepatotoxicity. Therefore, in the present study, we utilized RNA-sequencing and qRT-PCR validation to investigate CF-altered gene expression changes, ultimately identifying potential signaling pathways related to the regulatory mechanisms of CF toxicity to provide new insights into CF toxicology.

## 2. Results

### 2.1. Toxicological Study on CF-Induced Liver Injury

#### 2.1.1. Change in Biochemical Index

**Figure 1:**
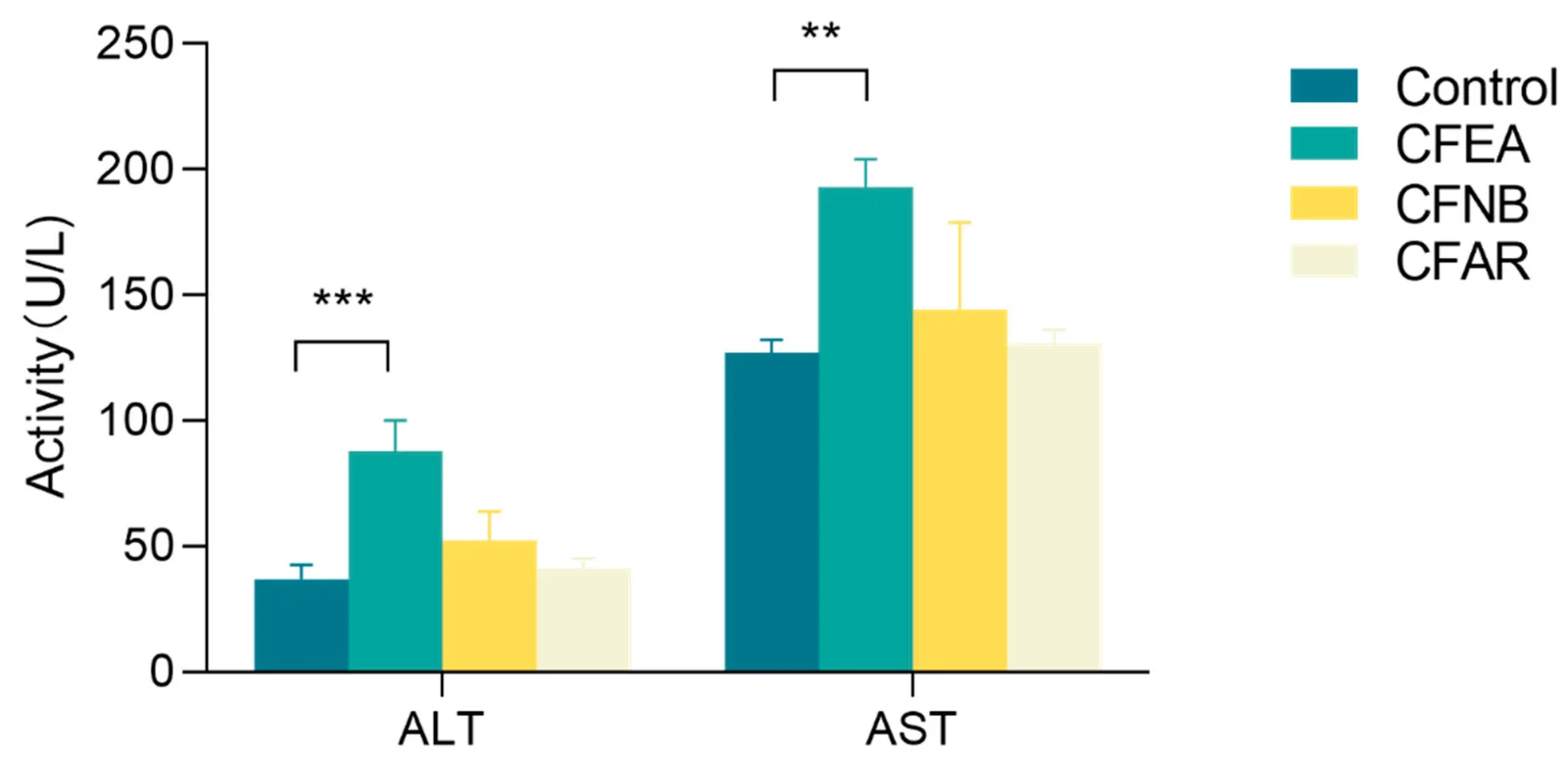
Blood chemistry indexes of liver injury: control, CFEA, CFNB and CFAR. Data are means ± SD, *n* = 3 for each bar. The statistically significant differences from the control were defined as, **, *p* < 0.01, ***, *p* < 0.001.

To verify the toxicity site of CF, we examined hematological and biochemical indicators. As shown in Figure 1, the data showed that CF ethyl acetate extract (CFEA) significantly increased serum AST and ALT levels (*p* < 0.05) compared with the con group, while CF n-butyl alcohol extract (CFNB) and CF aqueous residue extract (CFAR) had no significant differences (*p* > 0.05). In brief, these data suggest that CFEA can cause changes in hematological and biochemical parameters.

#### 2.1.2. Histopathological Morphology

**Figure 2:**
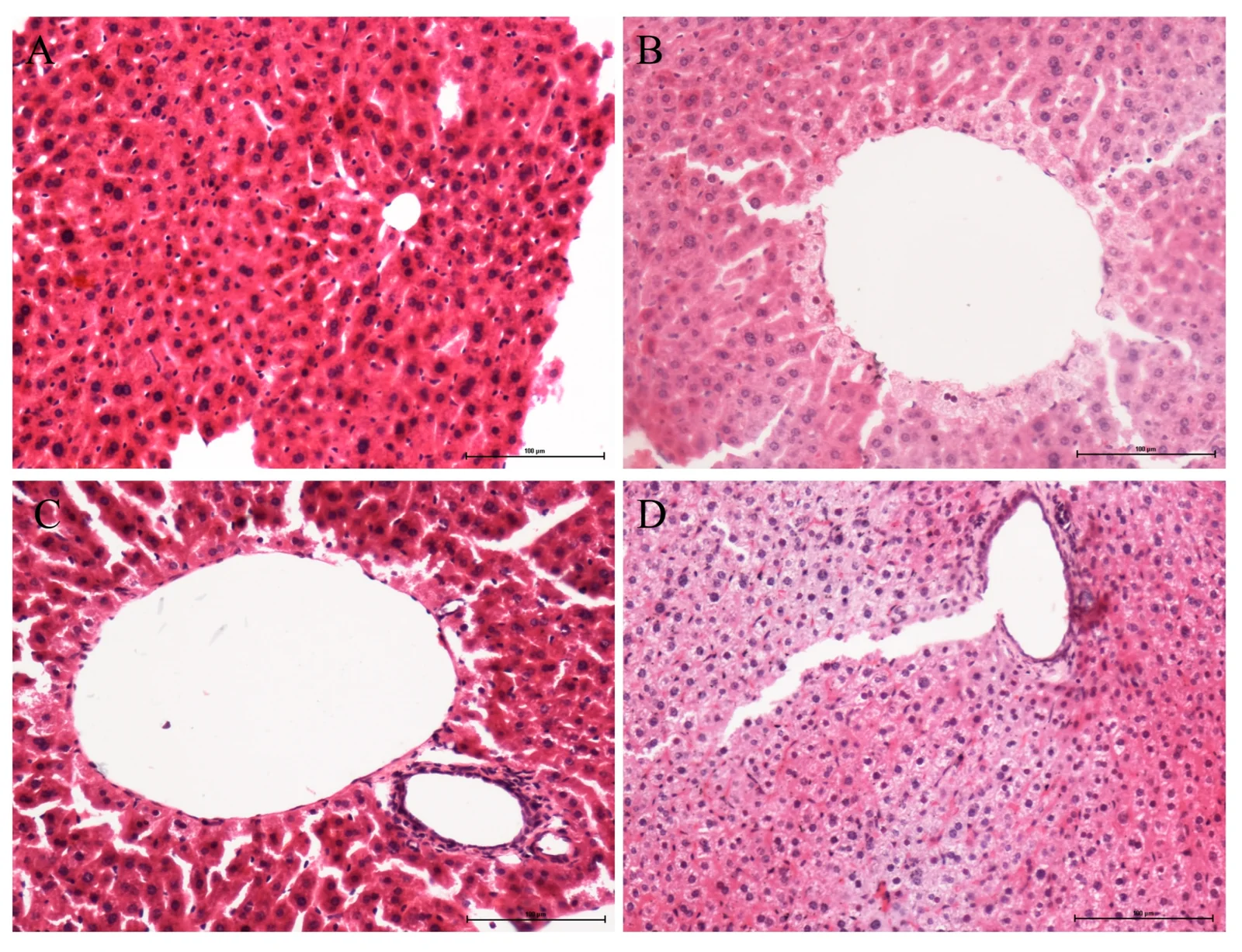
Micrograph of liver from male mice treated with water (**A**), CFEA (**B**), CFNB (**C**) and CFAR (**D**) 1 g/kg/d for 7 days. (**A**–**D**) Hepatic sections of the different groups stained with H&E (200×).

The liver sections of mice in the control group, CFNB and aqueous residue extract group showed the typical liver structure with the hepatocytes arranged radially around the central vein and well-formed (Figure 2A,C,D). The CFEA group showed vacuolar degeneration in the hepatocyte, as well as the nuclear lysis and cytoplasmic red staining (Figure 2B).

### 2.2. Transcriptome Sequencing

#### 2.2.1. Gene Ontology (GO) and Kyoto Encyclopedia of Genes and Genomes (KEGG) Functional Analysis in CF-Induced Differentially Expressed Genes (DEGs)

**Figure 3:**
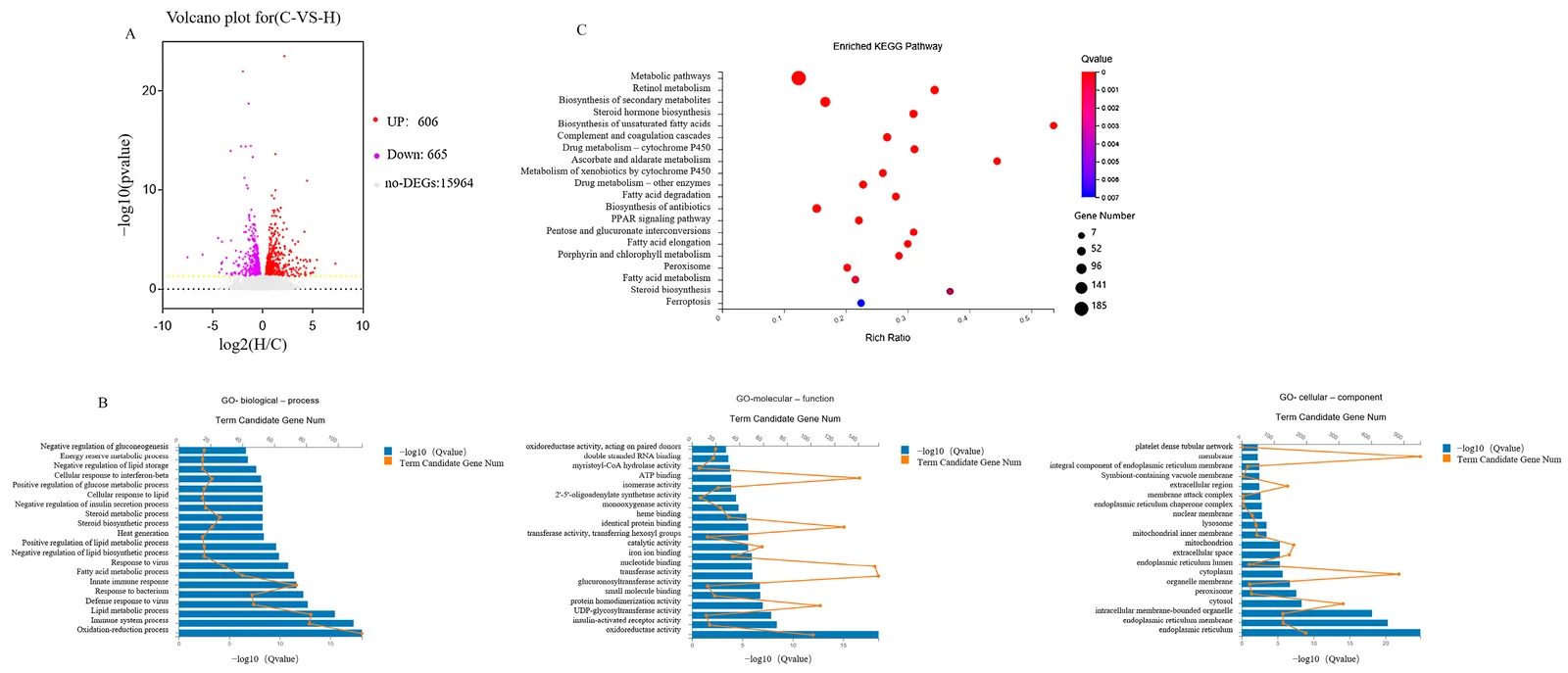
An analysis of DEG distribution ((**A**). volcano plot) between the control group and the CFEAt group. All gene expression levels are expressed as a log2 fold for CFEA group/Control group. (**B**). GO enrichment analysis (“Cellular component”, Molecular function” and “Biological process”). (**C**). KEGG assignment of unigenes in the Liver transcriptome of mice.

There were 1271 DEGs (606 up-regulated DEGs, red spots, and 665 down-regulated DEGs, blue spots) found according to |fold change| > 2 and *p*-adjustment < 0.05. Non-differentially expressed genes are represented by gray spots (Figure 3A).

DEGs were analyzed and grouped according to GO terms using David bioinformatics software (version 6.8). In Figure 3B, the top 20 GO terms with the highest DEG representation are listed in the molecular function, biological process, and cellular component categories. In the molecular function, the top three GO terms were oxidoreductase, insulin-activated receptor, and UDP-glycosyltransferase. The main GO terms included in the biological processes included immune system processes, the oxidation-reduction process and the lipid metabolic process. The GO terms mainly represented in the cellular components were endoplasmic reticulum, endoplasmic reticulum membrane and intracellular membrane-bounded organelle.

KEGG in DAVID bioinformatics software 6.8 was used to analyze the DEGs and resulted in 263 enriched KEGG pathways. Figure 3C shows the top 20 KEGG-enriched ones. Pathways related to lipid metabolism included biosynthesis of unsaturated fatty acids, fatty acid degradation, peroxisome proliferators-activated receptors (PPAR) signaling pathway and steroid hormone biosynthesis. Immune response-related pathways included the Complement and coagulation cascades. Cell growth and death-related pathway included Ferroptosis. Xenobiotics biodegradation and metabolism-related pathway included drug metabolism by cytochrome P450, metabolism of xenobiotics by cytochrome P450 and drug metabolism by other enzymes. This study was mostly focused on lipid metabolism-related pathways, especially the PPAR signaling pathway and steroid hormone biosynthesis.

#### 2.2.2. Lipid Metabolism Genes

**Figure 4:**
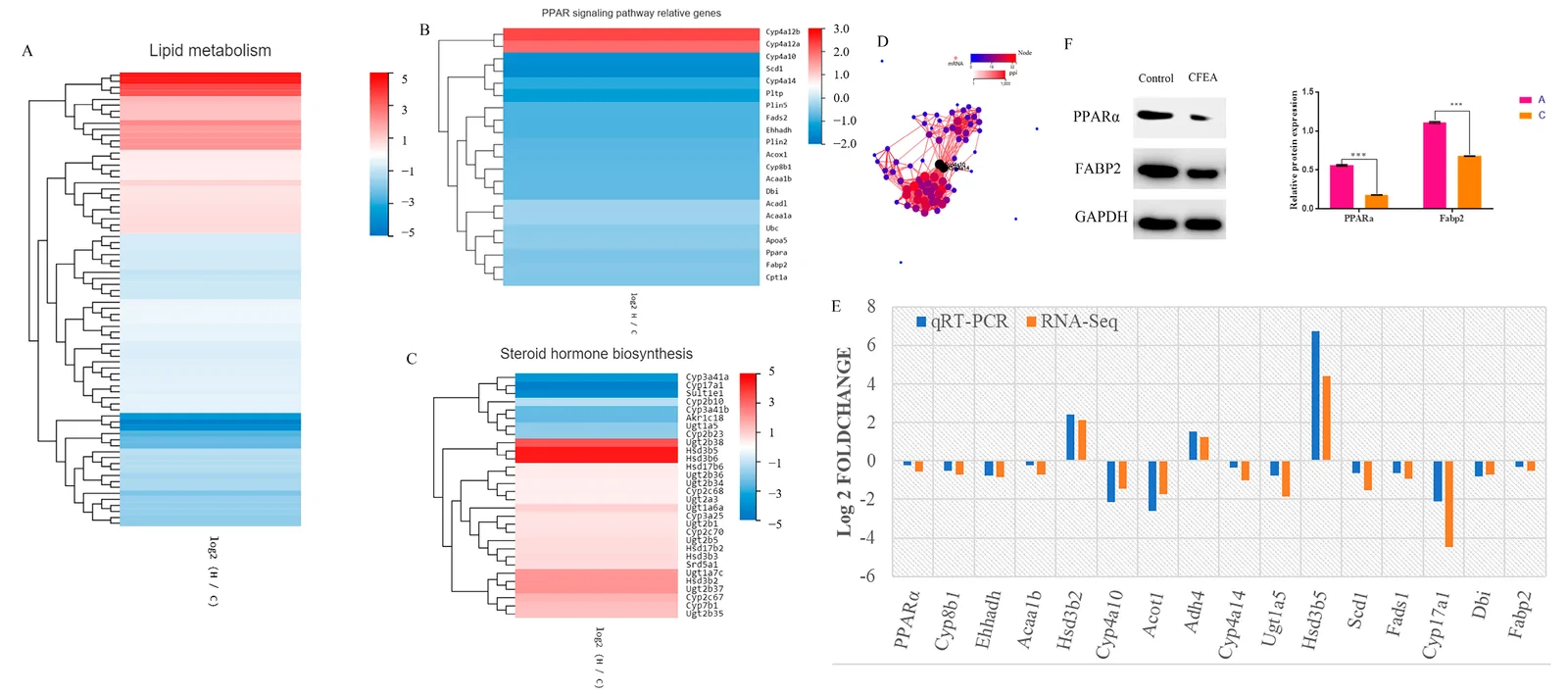
(**A**–**C**). Heatmap showing the relative gene expression of lipid metabolism pathway, PPAR signaling pathway and steroid hormone biosynthesis according to the results of RNA-seq. Log2 fold change is represented by the horizontal axis, and genes are represented by the vertical axis. Red indicates up-regulation, and blue indicates down-regulation in expression. (**D**). PPI network for 76 lipid metabolism proteins in the liver of mice with CF ethyl acetate extract treatment. (**E**). Up-regulated and down-regulated genes using RNA-Seq and qRT-PCR. (**F**). Protein levels of PPARα, Fabp2 as measured by Western blot. GAPDH was used as the control protein. Bars represent the mean ± SD. The statistically significant differences from the control were defined as ***, *p* < 0.001.

The liver performs important functions, including metabolism, bile production, detoxification, blood coagulation, and immunity processes. Since metabolism is essential in the development of hepatotoxicity, DEGs related to lipid metabolism were analyzed in detail after the administration of the CFEA group. According to RNA-seq results, 76 genes involved in the lipid metabolism pathway are shown as a heatmap (Figure 4A). Among them, 27 genes were up-regulated, and 49 were significantly down-regulated. CFEA induced a remarkable increase in the expression of Ugt2b38, Elovl3, Hsd3b6 and Hsd3b5 (>10-fold, *p* < 0.01) and a remarkable decrease in the expression of Cyp17a1, Sult1e1 and Cyp3a41a (>10-fold, *p* < 0.01).

#### 2.2.3. PPAR Signaling Pathway Genes

The liver also regulates fatty acids and cholesterol, as well as the energy and metabolism in the body, together with the adipose tissue. Figure 4B shows the list of the 21 genes in the CFEA group that were significantly dysregulated and involved in the PPAR signaling pathway that were mostly significantly decreased compared with the control group. Among them, 19 were related to lipid transport, fatty acid oxidation and transport, cholesterol metabolism and lipogenesis. For example, the treatment with the CFEA group significantly decreased the expression of PPARα (*p* = 0.00819) compared to the control group. This gene plays a key role in liver lipid metabolism. Cyp4a10 and Cyp4a14 significantly decreased, and these two genes are closely related to hepatic oxygen stress and lipid peroxidation of fatty acids, causing liver inflammation. Scd1, which regulates the balance of fatty acid metabolism and energy metabolism, was significantly decreased. The Cyp8b1 gene, which regulates the biosynthesis of bile acid, also significantly decreased.

#### 2.2.4. Steroid Hormone Biosynthesis Genes

An unexpected discovery was that the steroid hormone biosynthesis pathway was significantly enriched within the KEGG lipid metabolism pathway, which should be inactivated in the liver since it occurs in the adrenal glands and gonads. A comparison of CFEA and control groups shows 31 steroid hormone biosynthesis genes that are significantly altered by CFEA (Figure 4C). Indeed, the treatment in the CFEA group down-regulated the expression of Cyp17a1, Sult1e1, Cyp3a41a, Akr1c18, Cyp3a41b, Ugt1a5, Cyp2b23 and Cyp2b10, while it up-regulated the expression of Ugt2b34, Ugt2a3, Cyp2c68, Ugt2b36, Hsd17b6, Cyp3a25, Ugt2b1, Cyp2c70, Ugt2b5, Hsd17b2, Srd5a1, Hsd3b3, Ugt1a6a, Ugt2b35, Cyp7b1, Cyp2c67, Ugt1a7c, Hsd3b2, Ugt2b37, Ugt2b38, Hsd3b6 and Hsd3b5 compared with the expression of these genes in the control group.

Figure 4D shows the protein-protein interaction (PPI) network among 76 lipid metabolism proteins in the liver of mice after CFEA treatment. Cyp4a could be the key protein in the interaction of these pathways after its dysregulation by CFEA.

### 2.3. qRT-PCR

The expression of 16 DEGs related to lipid metabolism was confirmed using qRT-PCR, as shown in Figure 4E. Based on RNA-seq and qRT-PCR, the log2 (fold change) of each gene was calculated as follows: upstream genes such as Hsd3b2 (2.1 vs. 2.4), Adh4 (1.2 vs. 1.5) and Hsd3b5 (4.4 vs. 6.7) and downstream genes such as PPARα (0.6 vs. 0.2), Cyp8b1 (0.7 vs. 0.5), Ehhadh (0.9 vs. 0.8), Acaa1b (0.7 vs. 0.2), Cyp4a10 (1.4 vs. 2.1), Acot1 (1.7 vs. 2.6), Cyp4a14 (1.0 vs. 0.4), Ugt1a5 (1.9 vs. 0.8), Scd1 (1.5 vs. 0.7), Fads1 (0.9 vs. 0.6), Cyp17a1 (4.5 vs. 2.1), Dbi (0.7 vs. 0.8) and Fabp2 (0.5 vs. 0.3). According to these results, the trend in the expression of mRNA in these genes matched that found in the sequencing, suggesting that the results of the sequencing were accurate.

### 2.4. Western Blot (WB) of Key Proteins Related to the PPAR Signaling Pathway

The key proteins of PPARα and Fabp2 of the PPAR signaling pathway were evaluated by WB. The results showed that the phosphorylation of PPARα and Fabp2 was remarkably down-regulated in the CF ethyl acetate extract group compared to the control group (*p* < 0.01) (Figure 4F).

### 2.5. Identification of Constituents in the Active Fraction of the CFEA

**Figure 5:**
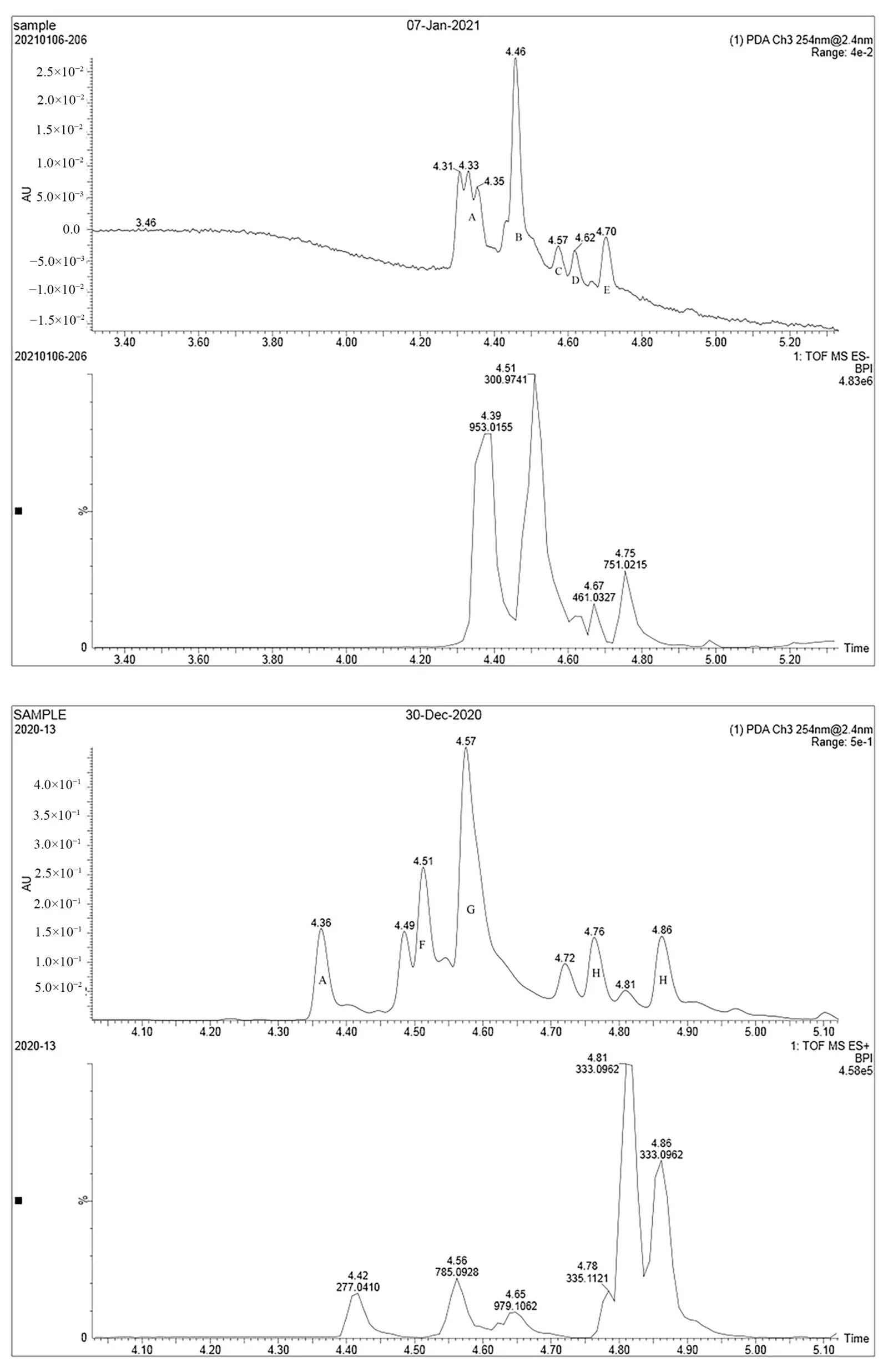
LC MS ion flow diagram. A–H stands for compound **A**–**H**.

The samples were analyzed using liquid chromatograph-mass spectrometer (LC-MS) technology in positive and negative ion scanning modes to determine the main components of CFEA. From the chemical spectrum of CFEA, the chemical composition in CFEA was deduced by comparison with the liquid chromatography and mass spectrometry of these compounds and with previously isolated substances that had been unambiguously identified [10] (Figure 5). As shown in Table 1, 8 compounds including 3,4,8,9,10-pentahydroxydibenzo [b,d]pyran-6-one (**A**, Pubchem ID: 341427420), ellagic acid (**B**, Pubchem ID: 5281855), 3′-*O*-methyl-4-*O*-(n″-*O*-galloyl-β-d-xylopyranosyl) ellagic acid (*n* = 2,3 or 4) (**C**), 3,3′-Di-*O*-methyl-4-*O*-(β-d-xylofuranosyl) ellagic acid (**D**, Pubchem ID: 14463056), 4-*O*-(3″,4″-*O*-digalloyl-α-l-rhamnosyl)ellagic acid (**E**), Terflavin B (**F**, Pubchem ID: 44584734), chebulic acid (**G**, Pubchem ID: 71308174) and galloylglucose (**H**, Pubchem ID: 124021) were identified. The MS spectra of each compound are shown in Supplementary Material Figures S1 and S2.

### 2.6. Results of Docking Studies

#### 2.6.1. PPARα Receptor Protein-Ligand Binding Position

**Figure 6:**
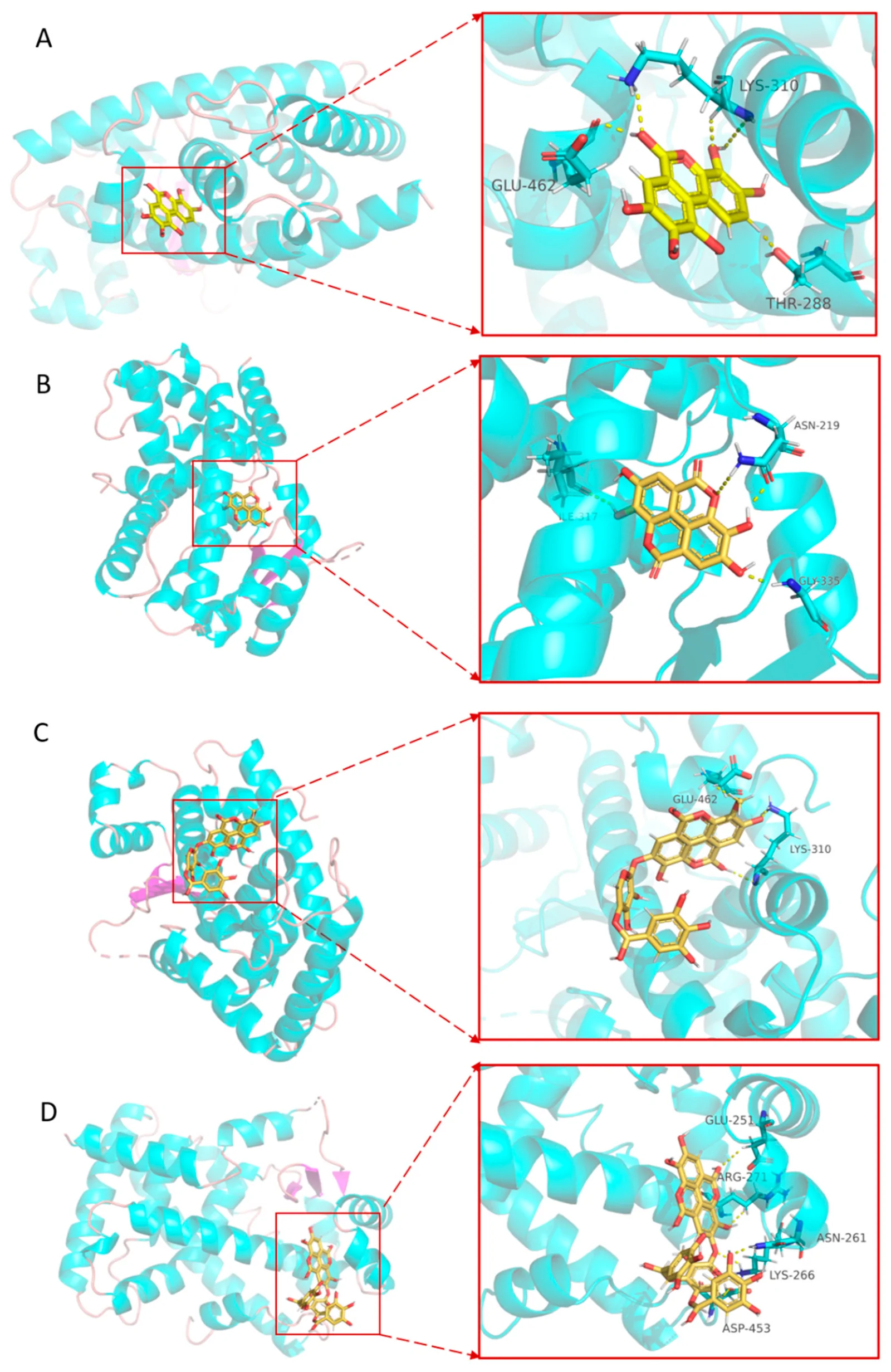
The molecular docking of four symbolic compounds with PPARα protein. 3,4,8,9,10-pentahydroxydibenzo [b,d]pyran-6-one (**A**), ellagic acid (**B**), 3′-*O*-methyl-4-*O*-(n″-*O*-galloyl-β-d-xylopyranosyl) ellagic acid (*n* = 2, 3 or 4) (**C**), and 4-*O*-(3″,4″-*O*-digalloyl-α-l-rhamnosyl) ellagic acid (**D**) with PPARα protein. The figure on the left shows that the compounds is located in the binding pocket of PPARα protein. The figure on the right shows the binding sites of compounds and PPARα protein.

Through molecular docking analysis, the interaction model of PPARα protein and CFEA compounds was determined at the molecular level. A lower binding affinity indicates a more stable binding of the two molecules. In this study, we docked the PPARα protein with eight active monomers of CFEA, respectively, and compared them with specific inhibitors (Table 2). The results showed that 3,4,8,9,10-pentahydroxydibenzo [b,d]pyran-6-one, ellagic acid, 3′-*O*-methyl-4-*O*-(n″-*O*-galloyl-β-d-xylopyranosyl) ellagic acid (*n* = 2, 3 or 4) and 4-*O*-(3″,4″-*O*-digalloyl-α-l-rhamnosyl) ellagic acid and PPARα protein binding energy are less than −6 kcal/mol, indicating good binding. Figure 6 shows the docking results of the four symbolic compounds with PPARα protein. The results showed that 3,4,8,9,10-pentahydroxydibenzo [b,d]pyran-6-one and 3′-*O*-methyl-4-*O*-(n″-*O*-galloyl-β-d-xylopyranosyl) ellagic acid (*n* = 2, 3 or 4) ligands bind to the receptor protein at the same amino acid of LYS310 as the inhibitor binding site. The two compounds interact with the amino acid residues of the protein mainly through hydrogen bonds (yellow dotted lines).

#### 2.6.2. FABP Receptor Protein-Ligand Binding Position

**Figure 7:**
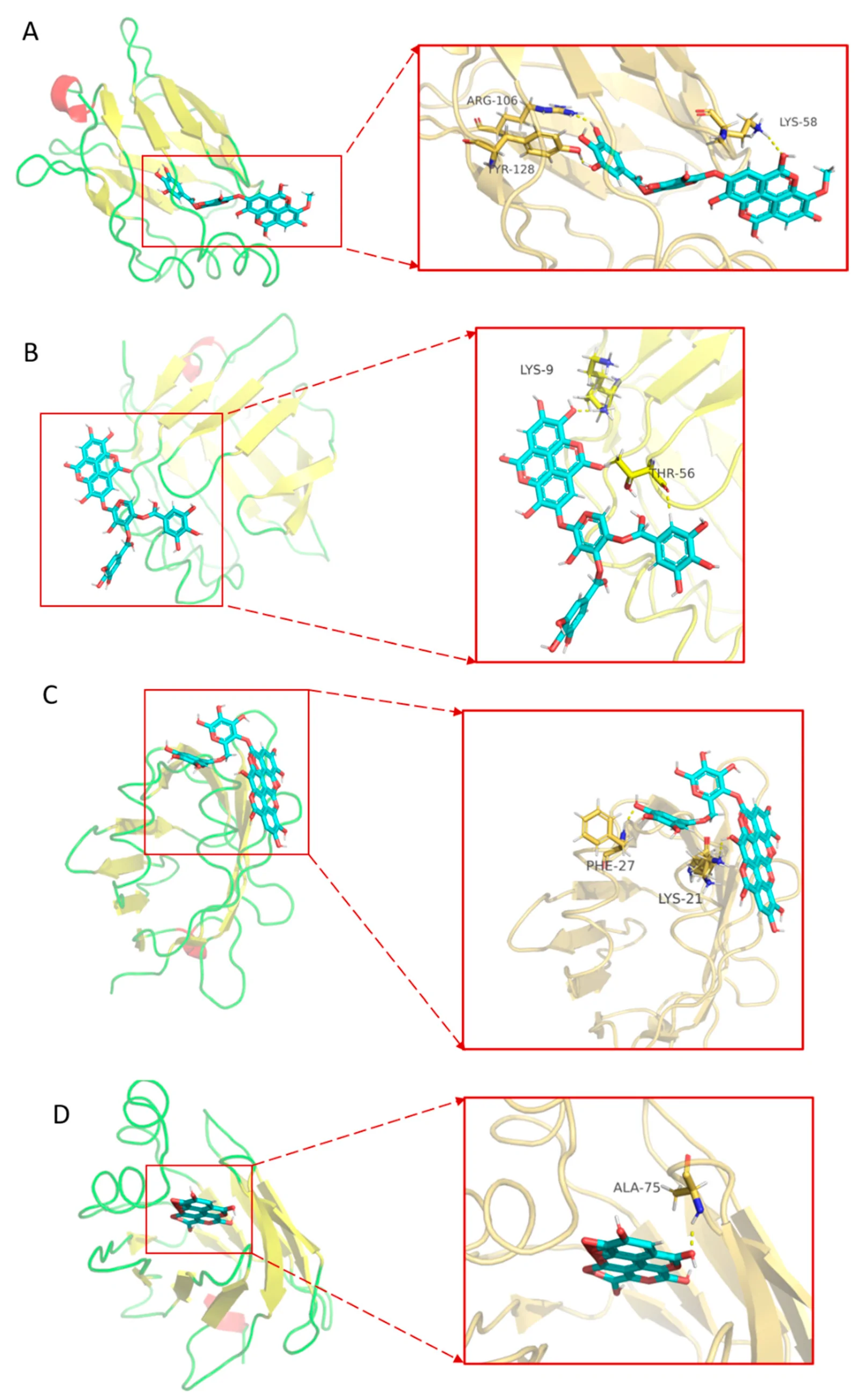
The molecular docking of four symbolic compounds with FABP protein. 3′-*O*-methyl-4-*O*-(n″-*O*-galloyl-β-d-xylopyranosyl) ellagic acid(*n* = 2, 3 or 4) (**A**), 4-*O*-(3″,4″-*O*-digalloyl-α-l-rhamnosyl)ellagic acid (**B**), terflavin B (**C**) and chebulic acid (**D**) with FABP protein. The figure on the left shows that the compounds is located in the binding pocket of FABP protein. The figure on the right shows the binding sites of compounds and FABP protein.

To further confirm the interaction between CFEA and FABP protein, the chemical components identified by CFEA were molecularly docked with the FABP protein. It was observed that in all compounds, the binding energies of 3′-*O*-methyl-4-*O*-(n″-*O*-galloyl-β-d-xylopyranosyl) ellagic acid (*n* = 2, 3 or 4), 4-*O*-(3″,4″-*O*-digalloyl-α-l-rhamnosyl) ellagic acid, Terflavin B and chebulic acid were less than −10 kcal/mo, which was −12.27, −10.65, −12.46 and −10.35, respectively (Table 3). Binding energy is a function of the stability of the complex formed between the ligand and target protein. Hydrogen bonds play an important role in the binding affinity of target drugs. For example, 3′-*O*-methyl-4-*O*-(n″-*O*-galloyl-β-d-xylopyranosyl) ellagic acid (*n* = 2, 3 or 4) forms three hydrogen bonds with FABP protein (Figure 7). These results indicate that 3′-*O*-methyl-4-*O*-(n″-*O*-galloyl-β-d-xylopyranosyl) ellagic acid (*n* = 2, 3 or 4), 4-*O*-(3″,4″-*O*-digalloyl-α-l-rhamnosyl) ellagic acid, terflavin B and chebulic acid are relatively stable in combination with FABP and are more likely to act on FABP protein.

## 3. Discussion

In the records of ancient Chinese literature and pharmacopeia, CF is described as being without any toxicity, but in 1985, some studies found that CF exerts evident hepatorenal toxicity [11]. Indeed, the level of serum urea, glucose and AST was significantly increased in the blood of Wistar rats after intragastric administration of 1 g/kg *Terminalia chebula Retz* for 28 days, suggesting a mild disturbance in liver and kidney function [9]. Our results are in agreement with these since CF ethyl acetate extract administered by gavage for 7 days at the dose of 1 g/kg resulted in a significant increase in ALT and AST levels, suggesting an injury in the liver that indeed was observed in the tissue sections.

The liver plays a major role in metabolism and is mainly responsible for the regulation of lipid metabolism. To better understand the mechanism of CF-induced hepatotoxicity, we treated mouse livers with 1 g/kg CF ethyl acetate extract for 1 week (in our study, this dose permanently caused significant liver damage without leading to death). KEGG enrichment analysis results showed that pathways related to lipid metabolism included Biosynthesis of unsaturated fatty acids, Fatty acid degradation, peroxisome proliferators-activated receptors (PPAR)PPAR signaling pathway, and Steroid hormone biosynthesis, which is the first type of pathway to be significantly affected. Disorders in lipid metabolism may be the cause of liver damage, such as fatty liver, chronic hepatitis and insulin resistance [12,13,14]. Xenobiotics biodegradation and the metabolism-related pathway included drug metabolism by cytochrome P450, metabolism of xenobiotics by cytochrome P450, and drug metabolism by other enzymes, which is the second type of pathway to be significantly affected. Cytochrome P450 2E1(CYP2E1) generates reactive metabolites inducing oxidative stress, mitochondrial dysfunction, and cell death [15]. There was a report that revealed that liver injury caused by EGCG and dieting might be related to the increase of pro-inflammatory lipid peroxidation products [16]. Meanwhile, the active components of CFEA have a similar structure to EGCG. Our results suggest that CF affects the expression of many genes related to lipid metabolism, and we speculate that the hepatotoxic mechanism of CFEA may be similar to that of EGCG, which is related to lipid metabolism.

A previous report revealed a significant correlation between steroid hormone biosynthesis and severe negative changes in energy balance in gene expression in the liver of cows [17]. Progesterone and adipoQ receptor 3 (PAQR3) promote the ubiquitination of PPARa through E3 ubiquitin ligase HUWE1, which negatively regulates the function of PPARa in vitro and in vivo [18]. Wilms’ tumor gene WT1 up-regulation increases the mRNA of CYP 17a1 and decreases progesterone secretion, likely by the inhibition of CYP 11a1 and 3β-Hsd [19]. In the present study, Hsd 3b (2, 3, 5, 6) expression was significantly reduced, and Cyp17a1 was significantly increased in mice treated with an oral administration of 1 g/kg CF ethyl acetate extract. In addition, our results showed that the expression of Ugt 2b (5, 34–38) was increased in C. Ugt enzyme combined with hydroxysteroid metabolites and pluronic acid produces more hydrophilic metabolites [20]. This is an important step in the removal of steroids since the accumulation of hydroxysteroid metabolites is due to the decrease in UGT, thus reducing the metabolism [21].

PPARα is a receptor and central regulator of hepatic lipid metabolism, expressed in the liver, skeletal muscle, and heart [22]. It regulates lipid metabolism by regulating fatty acid uptake, fatty acid activation and oxidation, lipolysis and ketogenic and triglyceride storage. Fatty acid binding protein (FABP) is one of the important regulatory proteins in lipid metabolism belonging to the lipid binding protein family [23]. L-FABP transports lipids to the nucleus and binds with PPARα, indirectly regulating fatty acids transport and absorption in hepatocytes, thus maintaining lipid homeostasis in the liver. Celastrol ameliorates acute liver injury through PPARα modulation [24]. Previously reported transcriptomic findings suggest that PPAR signaling is a latent key pathway for pyrazinamide-induced hepatotoxicity in mice [25]. The gene expression profiling in this study revealed that the PPAR signaling pathway was significantly enriched, and PPARα and FABP2 genes and proteins were significantly decreased in CF-treated mice compared with the control group. Thus, our hypothesis was that CF caused hepatotoxicity in mice through the L-FABP/PPARα pathway. Therefore, the PPARα agonist might be considered a potential therapeutic agent to combat the hepatotoxicity induced by CF. These results are similar to previous studies showing that PPAR-α-null mice cannot meet energy demands and develop hypoglycemia, hyperlipidemia, hypoketosis and fatty liver during fasting periods [26].

Molecular docking is a technology that can explore receptor-ligand binding modes and binding sites based on the structural docking calculation of target protein compounds and screen out the binding ability of certain chemical components to gene-encoded proteins. Molecular docking results showed that the binding site of the specific PPARα inhibitor GW6471 and the PPARα target protein was at the LYS310 amino acid. Among the chemical constituents identified from CFEA, 3,4,8,9,10-pentahydroxydibenzo [b,d]pyran-6-one, ellagic acid, 4-*O*-(3″,4″-*O*-digalloyl-α-l-rhamnosyl) ellagic acid and 3′-*O*-methyl-4-*O*-(n″-*O*-galloyl-β-d-xylopyranosyl) ellagic acid (*n* = 2, 3 or 4) bound well to the PPARα target protein. Meanwhile, when the chemical components identified by CFEA were docked with the FABP protein, it was found that 3′-*O*-methyl-4-*O*-(n″-*O*-galloyl-β-d-xylopyranosyl) ellagic acid (*n* = 2, 3 or 4), 4-*O*-(3″,4″-*O*-digalloyl-α-l-rhamnosyl) ellagic acid, Terflavin B and chebulic acid had lower binding energies to the FBBP target protein. Therefore, combined with the analysis of the two docking results, we predict that 3′-*O*-methyl-4-*O*-(n″-*O*-galloyl-β-d-xylopyranosyl) ellagic acid (*n* = 2, 3 or 4) and 4-*O*-(3″,4″-*O*-digalloyl-α-l-rhamnosyl) ellagic acid may inhibit the PPAR signaling pathway by inhibiting the PPARα protein and FABP protein, which may be the toxic components of CF.

## 4. Material and Methods

### 4.1. Chemicals and Animals

Ripe CF dried fruits were purchased from Harbin Shiyitang Traditional Chinese Medicine Pieces Co., Ltd., Harbin, China. It was identified by the Department of Traditional Chinese Medicine, Heilongjiang University of Chinese Medicine, Harbin, and a copy of the specimen has been submitted (voucher number 2009011ch). The dried CF (0.2 kg) was grounded and extracted by decoction for 1 h using distilled water (2.0 L) to obtain 21.3 g crude extract. The crude extract was suspended in water and extracted successively with ethyl acetate and n-butanol. The yields of ethyl acetate, n-butanol and residual water fraction obtained at last are 15.6%, 38.3% and 44.3%, respectively. Evaluate each fraction to screen for a hepatotoxic fraction. (All the extracts of CF were dried in a vacuum overnight, ensure it is without solvent).

A total of 40 7-week-old male ICR mice (initial body weight: 20 ± 2.0 g) were purchased from the Experimental Animal Center of Harbin Medical University. The feeding and environmental conditions were established by the regulations of the Animal Ethics Committee of Northeast Agricultural University and were approved by ethics (approval protocol No. SRM-06).

The male ICR mice were randomly divided into 4 groups: Control, CFEA, CFNB and CFAR. The final 3 groups (8 mice per group) were administered the different fractions of CF extract by gavage daily for a total of 1 week (1 g/kg/day). Control mice received the same amount of water. The mice were provided with adequate food and water during the entire experiment. After the last dose, mice were fasted overnight and anesthetized with halothane (1–1.5%) the next day. Abdominal vein blood collection (1.5–2 mL). After the mice were sacrificed by cervical dislocation, the livers were harvested, weighed, and divided into two parts: one was fixed in 10% neutral-buffered formalin for histopathology; the other was frozen in liquid N_2_ and stored at −80 °C for qRT-PCR, western blotting and RNA-Seq.

### 4.2. Biochemical Analysis

The level of alanine transaminase (ALT) and aspartate transaminase (AST) in the blood without anticoagulant was measured by an automatic clinical biochemical analyzer (Hitachi 7600).

### 4.3. Histological Analysis

Liver specimens were fixed in 4% formaldehyde for 24 h, dehydrated, embedded in paraffin, cut into 4 µm thick sections and stained with hematoxylin-eosin (H&E).

### 4.4. Transcriptome Sequencing

By the manufacturer’s instructions, total RNA was extracted from liver samples using Trizol reagent (CW0580S) [27]. Using NanoDrop and the Agilent 2100 bioanalyzer (Thermo Fisher Scientific, Waltham, MA, USA) for 6 samples (control group and CFEA group) detection of total RNA. Each sample was divided into 2 parts, one for RNA-Seq and the other one for qRT-PCR. A magnetic bead equipped with an Oligo(dT) attachment was used to purify mRNA. The first strand of cDNA was synthesized by segmenting the mRNA into small fragments using random hexamer primers. Synthesis of second strand cDNA with RNAseH and DNA polymerase I. The final library was amplified with phi29 to make a DNA nanoball (DNB) which had more than 300 copies of one molecular; DNBs were loaded into the patterned nanoarray, and single end 50 bases reads were generated on BGIseq500 platform (BGI-Shenzhen, China). The DEGs analysis were performed using the DESeq2(v1.4.5) with Fold Change > 2 and *p*-adjustment value < 0.05

In order to determine the DEGs’ functional and biological roles, GO terms and KEGG enrichment analysis were performed, and the pHYPER function of the R software 4.0 was used to determine the distribution function [28].

### 4.5. qRT-PCR

A total of 16 DEGs (PPARα, Cyp8b1, Ehhadh, Acaa1b, Hsd3b2, Cyp4a10, Acot1, Adh4, Cyp4a14, Ugt1a5, Hsd3b5, Scd1, Fads1, Cyp17a1, Dbi and Fabp2 purchased from TSINGKE, Beijing, China) were also measured by qRT-PCR using a LightCycler96 (Roche, Basel, Switzerland) to verify the reliability of the RNA-seq. Conditions were used as follows: initial denaturation at 95 °C for 600 s, 35 cycles of denaturation at 95 °C for 15 s and 30 s at 58 °C. The melting curve of each reaction at 65–95 °C was analyzed. GAPDH (Wanlei Biotechnology) was used as the internal standard. PrimeScript^TM^ RT reagent kit with gDNA Eraser (Takara Biomedical Technology (Beijing) Co., Ltd., Beijing, China) was used for the reverse transcription of the cDNA. The relative mRNA expression was calculated using the 2^−ΔΔCt^ method [29]. The primer sequences are listed in Table 4.

### 4.6. Western Blot

Western blot was used to measure the target proteins involved in the PPAR signaling pathway. Using RIPA lysis buffer (Beyotime, China, P00138), 100 mg of liver tissue (total protein) was extracted, and the concentration was determined using BCA protein reagent kits (Beyotime). The PVDF membrane was incubated overnight at 4 °C under shaking with primary antibodies against GAPDH (1:3000 dilution), Fabp2 (1:1000 dilution) and PPARα (1:1000 dilution). GAPDH and PPARα antibodies were purchased from Wanlei Biotechnology, Harbin, China. Fabp2 antibody was purchased from Proteintech Group, Inc. Detection of the bands was achieved using enhanced chemiluminescence (ECL) detection and quantitation using Image J software 1.8.0 after 1 h with anti-mouse and anti-rabbit IgG peroxidase (Wanlei Biotechnology, Harbin, China).

### 4.7. Analysis of Chemical Constituents of Ethyl Acetate Fraction of Myrobalan

Waters G2-XS Q-Tof mass spectrometry system and Waters Acquity Ultra-High Performance Liquid chromatography were used to analyze and identify the ethyl acetate extract of myrobalan. The column used was Waters Acquity UPLCBEH C18 (100 mm × 2.1 mm, 1.7 μm). The mobile phases were 0.1% formic acid aqueous solution (A) and methanol (B), the flow rate was 0.2 mL/min, the injection volume was 1 µL, the column temperature was 40 °C and the detection wavelength was 254 nm. The gradient elution program was 0–4 min, 5–28% B; 4–7 min, 28–40% B; 7–8 min, 40–60% B; 8–16 min, 60–100% B. The ESI ion source was used, the ion source temperature was 120 °C, the scanning mode was Positive TOF, the scanning range was 100–1200 Da, the capillary voltage was 2.5 kV, the desolvation gas temperature was 450 °C and the desolvation gas flow rate was 600 L/h. The cone gas flow rate was 0.63 L/h.

### 4.8. Molecular Docking Studies

The three-dimensional structure of the target protein (PPARα, PDB ID: 6KB7; FABP, PDB ID: 7FCX) was obtained from the RCSB protein database (PDB, http://www.rcsb.org, accessed on 10 February 2021), and the target protein was molecularly docked using AutoDock 1.5.6 software with chemical components identified by LC/MS and specific antagonists, respectively, running the auto grid with the grid box parameters set according to the inhibitor binding site. Docking by Lamarck Genetic Algorithm (LGA). The docking results were analyzed by the PLIP tool (https://plip-tool.biotec.tu-dresden.de/plip-web/plip/index, accessed on 10 February 2021), and the docking results of active compounds and protein targets were visualized.

### 4.9. Statistical Analysis

Statistical analysis was performed using SPSS (version 20.0). Volcano plots were created using GraphPad Prism (version 5.01). The transcriptome analysis was created using the BGI data mining website. Statistical significance was determined by *p* < 0.05 after comparing multiple groups using one-way analysis of variance (ANOVA).

## 5. Conclusions

This study evaluated the hepatotoxic effect of CF and the related molecular mechanism by transcriptome analysis and network toxicology. The results showed that DEGs are involved in lipid metabolism and, unexpectedly, also in the biosynthesis of steroid hormones, a process not normally performed by the liver. In addition, 4-*O*-(3″,4″-*O*-digalloyl-α-l-rhamnosyl) ellagic acid and 3′-*O*-methyl-4-*O*-(n″-*O*-galloyl-β-d-xylopyranosyl) ellagic acid (*n* = 2, 3 or 4) may be the main chemical component of CFEA that inhibit PPARα protein and FABP protein. The PPAR signaling pathway was found as the key pathway involved in the hepatotoxicity of CF. Therefore, this study provides a new perspective in the evaluation of the toxic effect and mechanisms of traditional Chinese herbal medicines.

## Figures and Tables

**Table 1:** HPLC–ESI-MS data of the major compounds of CFEA.

Compounds	R_t_ (min)	Chemical Structure
A	4.35	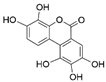
B	4.46	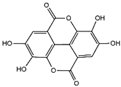
C	4.57	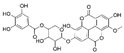
D	4.62	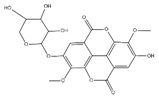
E	4.70	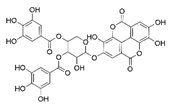
F	4.51	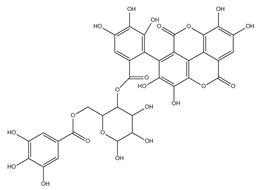
G	4.57	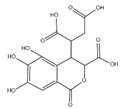
H	4.76, 4.86	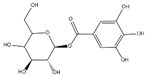

**Table 2:** PPARα receptor protein-ligand binding position.

Compounds	Binding Site	Hydrogen Bonds	Binding Affinity (kcal/mol)
GW6471	LYS310	1	−7.00
3,4,8,9,10-pentahydroxydibenzo [b,d]pyran-6-one	THR288 GLU462 LYS310	5	−6.64
ellagic acid	ILE317 ASN219 GLY335	4	−7.51
3′-*O*-methyl-4-*O*-(n″-*O*-galloyl-β-d-xylopyranosyl) ellagic acid (*n* = 2, 3 or 4)	GLU462 LYS310	3	−8.10
3,3′-Di-*O*-methyl-4-*O*-(β-d-xylofuranosyl) ellagic acid	GLY296 PHE297 ALA298 ASN299	4	−3.57
4-*O*-(3″,4″-*O*-digalloyl-α-l-rhamnosyl) ellagic acid	GLU251 ASN261 LYS266 ARG271 ASP453	5	−6.3
Terflavin B	GLU289 THR288 GLU462 LYS310 LYS292	6	−3.07
chebulic acid	GLU369 ARG226 SER230	4	−2.06
galloylglucose	VAL306 LYS310 GLU462	3	−2.83

**Table 3:** FABP receptor protein-ligand binding position.

Compounds	Binding Site	Hydrogen Bonds	Binding Affinity (kcal/mol)
3,4,8,9,10-pentahydroxydibenzo [b,d]pyran-6-one	THR60 TYR128	2	−9.55
ellagic acid	LYS21 THR121	3	−8.29
3′-*O*-methyl-4-*O*-(n″-*O*-galloyl-β-d-xylopyranosyl) ellagic acid (*n* = 2, 3 or 4)	LYS58 ARG106 TYR128	3	−12.27
3,3′-Di-*O*-methyl-4-*O*-(β-d-xylofuranosyl) ellagic acid	LYS9	1	−9.37
4-*O*-(3″,4″-*O*-digalloyl-α-l-rhamnosyl) ellagic acid	LYS9 THR56	2	−10.65
Terflavin B	PHE27 LYS21	2	−12.46
chebulic acid	ALA75	1	−10.35
galloylglucose	ARG106	1	−9.08

**Table 4:** Primers used for qRT-PCR.

Gene	Primers Sequences	Fragment Length	Accession Number
PPARα (F)	ACGATGCTGTCCTCCTTGATGAAC	108	NM_001113418
PPARα (R)	GATGTCACAGAACGGCTTCCTCAG		
Cyp8b1 (F)	ACACCAAGGACAAGCAGCAAGAC	130	NM_010012.3
Cyp8b1 (R)	TGGCTCACTTCCACCCACTCC		
Ehhadh(F)	CGTCTCCTCGGTTGGTGTTCTTG	110	NM_023737.3
Ehhadh (R)	GCTGCTTTGGGTCTGACTCTACAG		
Acaa1b (F)	CAAGGCAGGTTGTCACGCTACTC	105	NM_146230.3
Acaa1b (R)	AGACCGCAGCAGCTCCCATC		
Hsd3b2 (F)	GTGTCATTCCCAGGCAGACCATC	88	NM_001359741.1
Hsd3b2 (R)	GCTGGCACACTGGCTTGGATAC		
Cyp4a10 (F)	TCTGTGCTCGGTCTGCTCCTG	97	NM_010011.3
Cyp4a10 (R)	GAGGTGATGGGAACTGCTGGAAAG		
Acot1 (F)	GCTGGCTGGGAAGGGCTTTG	117	NM_012006.2
Acot1 (R)	CGCAGGTAGTTCACGGCTTCTTC		
Adh4 (F)	AGGCAAACTTGGAGAGAGTGTGTC	84	NM_011996.2
Adh4 (R)	GGGTGACCTTGGCAGTATTGATGG		
Cyp4a14 (F)	AGCTACCAAGGCAGTGTTCAGTTG	95	NM_007822.2
Cyp4a14 (R)	TTCCGCAGGCGAAAGAAAGTCAG		
Ugt1a5 (F)	GACTCGGGCATTCATCACACACTC	81	NM_201643.2
Ugt1a5 (R)	GGCATCATCACCATCGGAACTCC		
Hsd3b5 (F)	GGCATCATCACCATCGGAACTCC	124	NM_008295.2
Hsd3b5 (R)	TGAATGTTGGCACACTGGCTTCC		
Scd1 (F)	AGCCTGTTCGTTAGCACCTTCTTG	143	NM_009127.4
Scd1 (R)	GCACCCAGGGAAACCAGGATATTC		
Fads1 (F)	TCCTGGTCTACCTGCTTCACATCC	133	NM_146094.2
Fads1 (R)	CAACCTGCCTGAGCCTGAACTG		
Cyp17a1 (F)	GTCACGGTGGGAGACATCTTTGG	149	NM_007809.3
Cyp17a1 (R)	GACGGTGTTCGACTGAAGCCTAC		
Dbi (F) (R)	CAAAGCCGCTGAGGAGGTGAAG	96	NM_001037999.2
Dbi (R)	TCGCCCACAGTAGCTTGTTTGAAG		
Fabp2 (F)	GCTGATTGCTGTCCGAGAGGTTTC	82	NM_007980.3
Fabp2 (R)	AAAGAATCGCTTGGCCTCAACTCC		

## Data Availability

The data presented in this study are available in the manuscript.

## Supplementary Materials

The following supporting information can be downloaded at: https://www.mdpi.com/article/10.3390/molecules28083427/s1, Figure S1: Compound MS spectra of Negative Ion Scanning: 3,4,8,9,10-pentahydroxydibenzo [b,d]pyran-6-one (A), ellagic acid (B), 3′-*O*-methyl-4-*O*-(n″-*O*-galloyl-β-d-xylopyranosyl) ellagic acid (*n* = 2, 3 or 4) (C), 3,3′-*O*-Dimethyl-4-*O*-(β-d-xylofuranosyl) ellagic acid (D), 4-*O*-(3″,4″-*O*-digalloyl-α-l-rhamnosyl)ellagic acid (E). Figure S2: Compound MS spectra of Positive Ion Scanning: 3,4,8,9,10-pentahydroxydibenzo [b,d]pyran-6-one (A), Terflavin B (F), chebulic acid (G), and galloylglucose (H).
