# Correction: Ai et al. Hepatotoxic Components Effect of Chebulae Fructus and Associated Molecular Mechanism by Integrated Transcriptome and Molecular Docking. *Molecules* 2023, *28*, 3427

**DOI:** 10.3390/molecules31173053

**Published:** 2026-08-31

**Authors:** Liwen Ai, Fan Yang, Wanjun Hu, Liyang Guo, Weixue Liu, Xuexue Xue, Lulu Li, Zunlai Sheng

**Affiliations:** 1 College of Veterinary Medicine, Northeast Agricultural University, Harbin 150030, China; ailiwen0228@163.com (L.A.); yangfan19970708@163.com (F.Y.); huwanjun1120@163.com (W.H.); 1884617844@163.com (L.G.); qwer13763688594@163.com (W.L.); xuexuexue0359@sina.com (X.X.); liluluzs@163.com (L.L.); 2 Heilongjiang Key Laboratory for Animal Disease Control and Pharmaceutical Development, Harbin 150030, China

## Error in Figure

In the original publication [1], there was a mistake in Figure 2 as published. Figures 2A and 2C have been incorrectly used. The corrected Figure 2—showing a micrograph of liver from male mice treated with water (A), CFEA (B), CFNB (C), and CFAR (D) 1 g/kg/d for 7 days, as well as hepatic sections of the different groups stained with H&E (200×)—appears below. The authors state that the scientific conclusions are unaffected. This correction was approved by the Academic Editor. The original publication has also been updated.

## Figures and Tables

**Figure 2:**
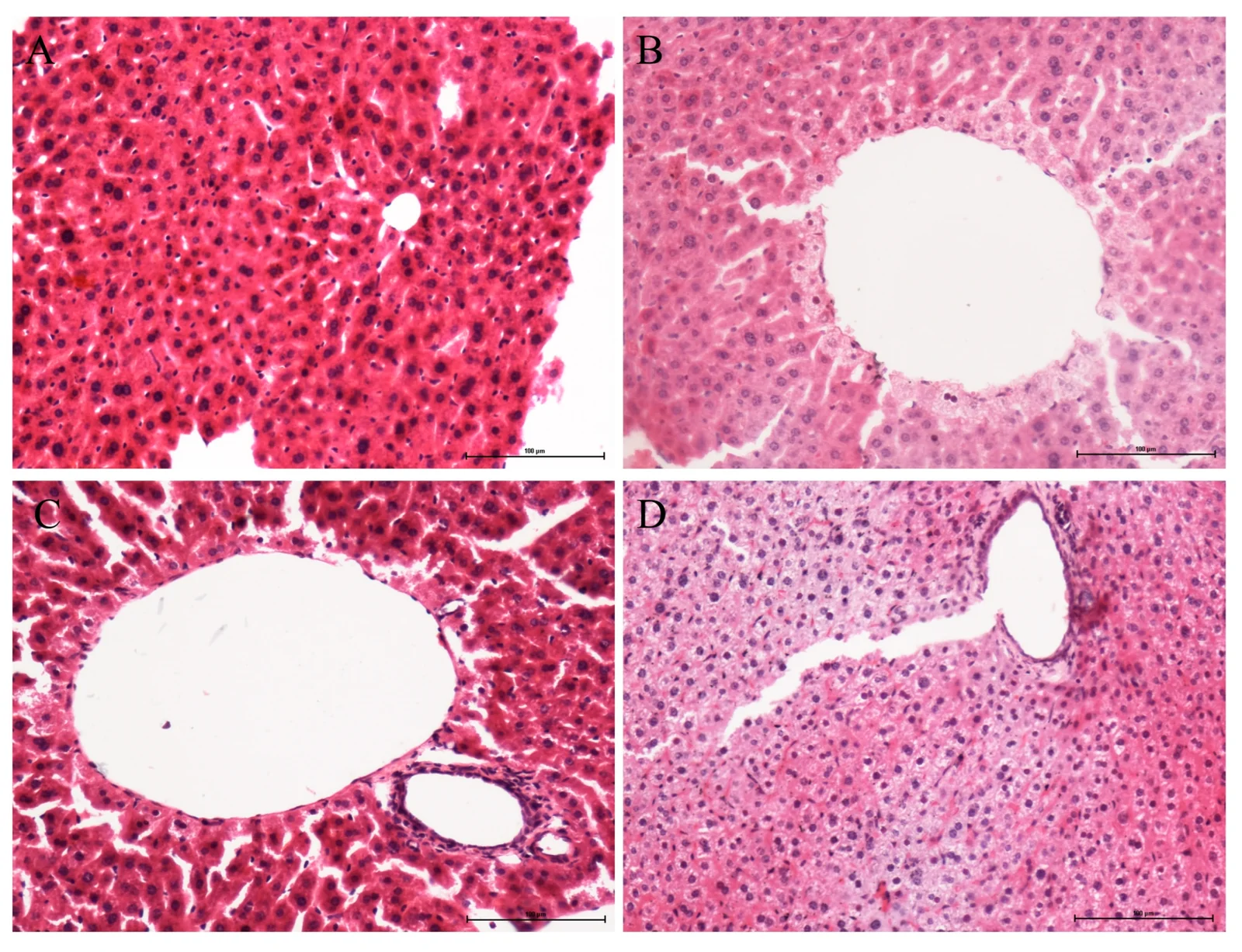
Micrograph of liver from male mice treated with water (**A**), CFEA (**B**), CFNB (**C**) and CFAR (**D**) 1 g/kg/d for 7 days. (**A**–**D**) Hepatic sections of the different groups stained with H&E (200×).
