# Supplementary material for: Hepatotoxic Components Effect of Chebulae Fructus and Associated Molecular Mechanism by Integrated Transcriptome and Molecular Docking
Source: Molecules. 2023 Apr 13;28(8):3427. doi: 10.3390/molecules28083427 (PMC10143891; doi:10.3390/molecules28083427)
Supplement: Supplementary file 1 [file molecules-28-03427-s001.zip › molecules-2288581-supplementary.pdf]

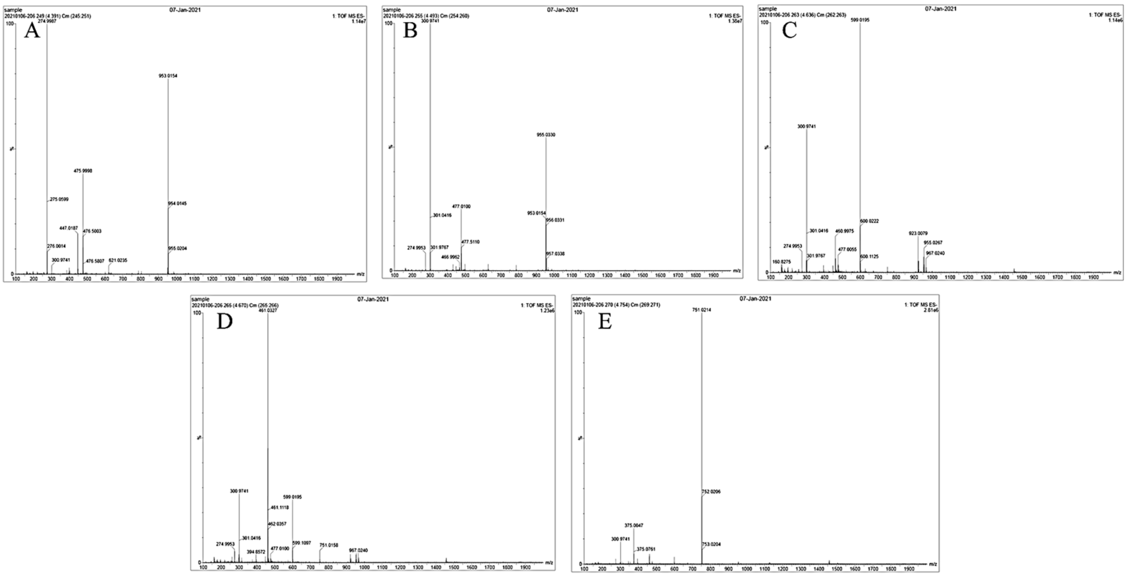

Figure S1 Compound MS spectra of Negative Ion Scanning：3,4,8,9,10-pentahydroxydibenzo [b,d]pyran-6-one(A), ellagic acid(B), 3'-O-methyl-4-O-(n'' -O-galloyl-β-D-xylopyranosyl) ellagic acid(n=2,3 or 4)(C), 3,3'-O-Dimethyl-4-O-(β-d-xylofuranosyl) ellagic acid(D), 4-O-(3'',4''-O-digalloyl-α-l-rhamnosyl)ellagic acid(E)

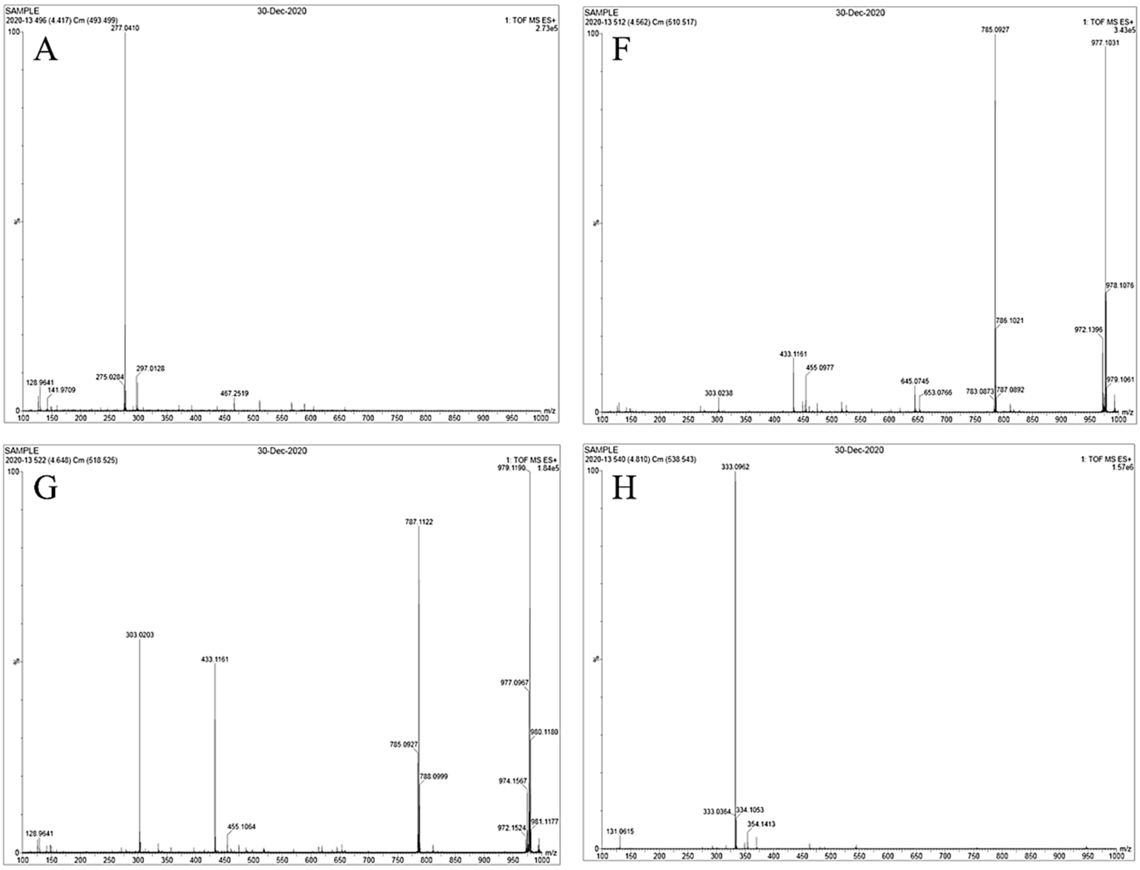

Figure S2 Compound MS spectra of Positive Ion Scanning：3,4,8,9,10-pentahydroxydibenzo [b,d]pyran-6-one(A), Terflavin B(F), chebulic acid(G), and galloylglucose(H).
